# Hearing rehabilitation and microbial shift after middle ear surgery with Vibrant Soundbridge in patients with chronic otitis media

**DOI:** 10.1007/s00405-022-07795-9

**Published:** 2023-01-20

**Authors:** Karl-Ludwig Bruchhage, Mariia Lupatsii, Friederike Möllenkolk, David Leffers, Arwa Kurabi, Tim Jürgens, Simon Graspeuntner, Daniela Hollfelder, Anke Leichtle

**Affiliations:** 1 Department of Otorhinolaryngology, Head and Neck Surgery, University Hospital Schleswig-Holstein, Lübeck, Germany; 2 Department of Infectious Diseases and Microbiology, University Hospital Schleswig-Holstein, Lübeck, Germany; 3 Division of Otolaryngology, Department of Surgery, UCSD School of Medicine, La Jolla, San Diego, USA; 4 German Center for Infection Research (DZIF), Partner Site Hamburg-Lübeck-Borstel-Riems, Hamburg, Germany; 5 Institute of Acoustics, University of Applied Sciences Lübeck, Lübeck, Germany

**Keywords:** Otitis media, Hearing restoration, Quality of life, Microbiome, Microbial colonization, Vibrant Soundbridge, VSB, Active middle ear implant, Hearing loss

## Abstract

**Introduction:**

Patients with otitis media (OM) encounter significant functional hearing impairment with conductive, or a combined hearing loss and long-term sequelae involving impaired speech/language development in children, reduced academic achievement and irreversible disorders of middle and inner ear requiring a long time therapy and/or multiple surgeries. In its persistent chronic form, Otitis media (COM) can often only be treated by undergoing ear surgery for hearing restoration. The persistent inflammatory reaction plays a major role, often caused by multi-resistant pathogens in the ear. Herein, we present outcomes of patients implanted with currently the only FDA approved active Middle Ear Implant Vibrant Soundbridge (VSB), suffering from persistent COM.

**Methods:**

The study enrolled 42 patients, treated by performing middle ear (ME) surgery to different extents and implanted with the VSB to various structures in the ME. Included were 17 children and 25 adults that had recurrent and/or persisting OM and significant hearing loss. Preoperative and postoperative patients' audiometric data were evaluated and the benefit with VSB assessed using the Glasgow Benefit Inventory for adults and pediatric cohorts. The microbial spectrum of pathogens was assessed before and after surgery, exploring the colonization of the otopathogens, as well as the intestinal microbiome from individually burdened patients.

**Results:**

The mean functional gain is 29.7 dB HL (range from 10 to 56.2 dB HL) with a significant improvement in speech intelligibility in quiet. Following VSB implantation, no significant differences in coupling were observed at low complication rates. Postoperatively patients showed significantly increased benefit with VSB compared to the untreated situation, including less otorrhea, pain, medical visits, and medication intake, with no recurrent OM and significant bacterial shift in otopathogens. The analysis of the intestinal microbiome displayed a high abundance of bacterial strains that might be linked to chronic and persistent inflammation.

**Conclusions:**

Functional ear surgery including rehabilitation with a VSB in patients suffering from COM present to be safe and effective. The successful acceptance accompanied by the improved audiological performance resulted in significant benefit with VSB, with a shift in the ear pathogens and altered microbiome and thus is a great opportunity to be treated.

## Introduction

Otitis media (OM) is one of the diseases with a significantly high socio-economic burden on the healthcare system [1, 2]. In industrialized nations, OM is the leading cause for physician visits, antibiotic prescriptions, and operations [3], while it is considered a major health problem due to high child mortality rates in developing countries [1]. The causes are multifactorial, immunodeficiency and environmental influences plus familial and genetic disposition were associated with increased prevalence of chronic inflammation [2]. Clinically, the microbial colonization and its virulence seem to regulate infection pathogenesis [2] and promote the formation of biofilms in the middle ear (ME) mucosa [4]. In therapy-resistant chronic otitis media (COM), two pathogens are particularly prevalent: *Pseudomonas aeruginosa* and *Staphylococcus aureus* [5, 6]. Both trigger a highly inflammatory ME response reflected by significantly increased presence of inflammatory markers, such as TNF and IL8, IL1β and other cytokines [7, 8]. In cholesteatoma disease, microbial pathogenicity manifests itself in a locally destructive inflammation-triggered epicenter, leading to a superinfected "blow out" cholesteatoma [9]. The etiopathogenesis of this is still unclear, but has been explained through chronic, persistent ME infections via the immune response of the innate immune system [10, 11] and the bacterial colonization of the retraction pocket with increased proliferation [12]. The bacteria play a role in inciting increased bone destruction through degrading osteoclasts in cholesteatoma by means of microbial Lipopolysaccharides (LPS) that trigger pro-inflammatory responses through the TLR4–MyD88 immune mechanisms [13]. Involvement of other innate immune receptors such as the TLRs and NLRs in cholesteatoma confirm this theory of inflammation-triggered growth through immune signaling pathways [10, 14]. One explanation is that these pathogens escape detection by the immune system through their ability to form biofilms [15]. A more recent theory is the “persister cell” formation of pathogens in the ME [9, 15]. These "persister cell" pathogens, a polymicrobial community made up of aerobic and anaerobic bacteria, classically *P. aeruginosa* and *S. aureus* [10], are able to survive without biofilm and evade the immune system and antibiotic therapies [9].

Since microbial infections, especially bacterial colonization and tissue biofilms, play a major role in the development and chronification of OM (15, 9, 4–6), the current guidelines for COM therapy recommend antibiotics as a first-line treatment, such as ear drops, oral or intravenous. However, often no significant improvements are marked [2, 16]. On the contrary, more local resistances and intolerances are formed in some cases [2]. In this context, it is of increasing interest to investigate not only the local bacterial colonization, but also the intestinal microbiome in particular, since it may be closely related to the regulation of various inflammatory processes [17, 18]. For example, in mice, a tryptophan-deficient diet lead to an imbalance in the intestinal flora and an increased susceptibility to systemic inflammation [19].

COM leads to significant functional impairments with significant effects on quality of life. The symptoms are characterized by otorrhea, hearing loss, delayed speech development up to complete deafness and dizziness [20, 21]. This is particularly evident in the course of specifically aggressive COM and cholesteatoma (chronic OM epitympanalis). Cholesteatoma behaves like a malignant tumor; it evades antibiotic therapy, the proinflammatory response is destructive to the ME and progressively destroys the surrounding structures [9, 15, 22, 23]. Recurrent inflammation can only be managed through appropriate surgical rehabilitation with complete removal of the inflammation foci [24] to preserve the important structures such as the inner ear (IE), the vestibular system, the facial nerve and prevent further otogenic complications, such as meningitis or brain abscess, which would otherwise be fatal [9, 15, 22]. The aim is to preserve hearing, which if primarily not possible takes place secondarily to healing by means of classic hearing structures or an active middle ear implant (Vibrant Soundbridge). In the case of implantation, it is of the utmost importance to have an inflammation-free middle ear and mastoid cavity.

Introduced in the late 90’s, the active ME implant Vibrant Soundbridge (VSB) from Med-El, has been used for hearing rehabilitation in patients with mild to severe sensorineural hearing loss unable to tolerate conventional hearing aids (HAs). VSB can also be used in the treatment of conductive or mixed hearing loss with a suitable vibratory structure to benefit from amplification, as for reconstruction of the hearing after cholesteatoma or ear surgery with good benefit. The system is indicated for both, children and adults [25–27]. Various authors showed the efficacy and safety of the VSB in the past decade in a number of studies [25–37], and was systematically evaluated in several review articles [38–40].

This study aimed to investigate how ME surgery followed by hearing rehabilitation, using the VSB implant in patients suffering from severe COM, can be utilized to overcome recurrent inflammation and associated chronification with hearing impairment due to microbial ME infections through selective treatment. As well, we evaluated the bacterial colonization and the intestinal microbiome.

## Methods

### Study design and sample collection

Study subjects were patients undergoing surgical treatments, to various levels, due to COM between June 2014 and August 2021. All suffered from a moist middle ear or radical cavity. Reconstruction with a partial (Porp) or full prothesis (Torp) did not have the desired effect. The wearing of a conventional hearing aid (HA) prior to the visit in our clinic was in the majority of the subjects performed (73%) and documented, led to recurrent or persistent otorrhea, hearing impairment and persistent therapy resistant microbial infections of the ME.

Medical examinations, sample collection and surgical treatments were performed at the Department of Otorhinolaryngology, University Hospital Schleswig–Holstein, Lübeck Campus. All patients gave written and informed consent. The study was approved by the institutional ethics committee at the University of Lübeck (AZ20–019 and 10–039), and conducted in accordance with the ethical principles for medical research formulated in the WMA Declaration of Helsinki.

A total of 42 patients (19 female, 23 male) were considered with an overall mean age of 33.1 years (SD ± 20.5 years; ranged from 6 to 80 years) at the initial ear surgery occurrence. They were 4 years and 6 months on average later implanted with an active transcutaneous middle ear implant Vibrant Soundbridge (VSB, MED-El, Innsbruck, Austria), either in the left (*n* = 18) or the right (*n* = 24) ear. Dependent on the anatomical structures of the ME, the surgeon considered the most efficient coupling. With regard to the ear surgery age range, the patients were divided into adults (*n* = 25; 46.9 years SD ± 14.7 years) and children (*n* = 17; 12.8 years ± 3.19 years); all with varied degrees of hearing loss and limitation in the use and efficacy of conventional hearing aids (HA). All patients suffered from long-term otorrhea and hearing impairment due to COM, most of which started in childhood.

### Surgery

**Fig. 1:**
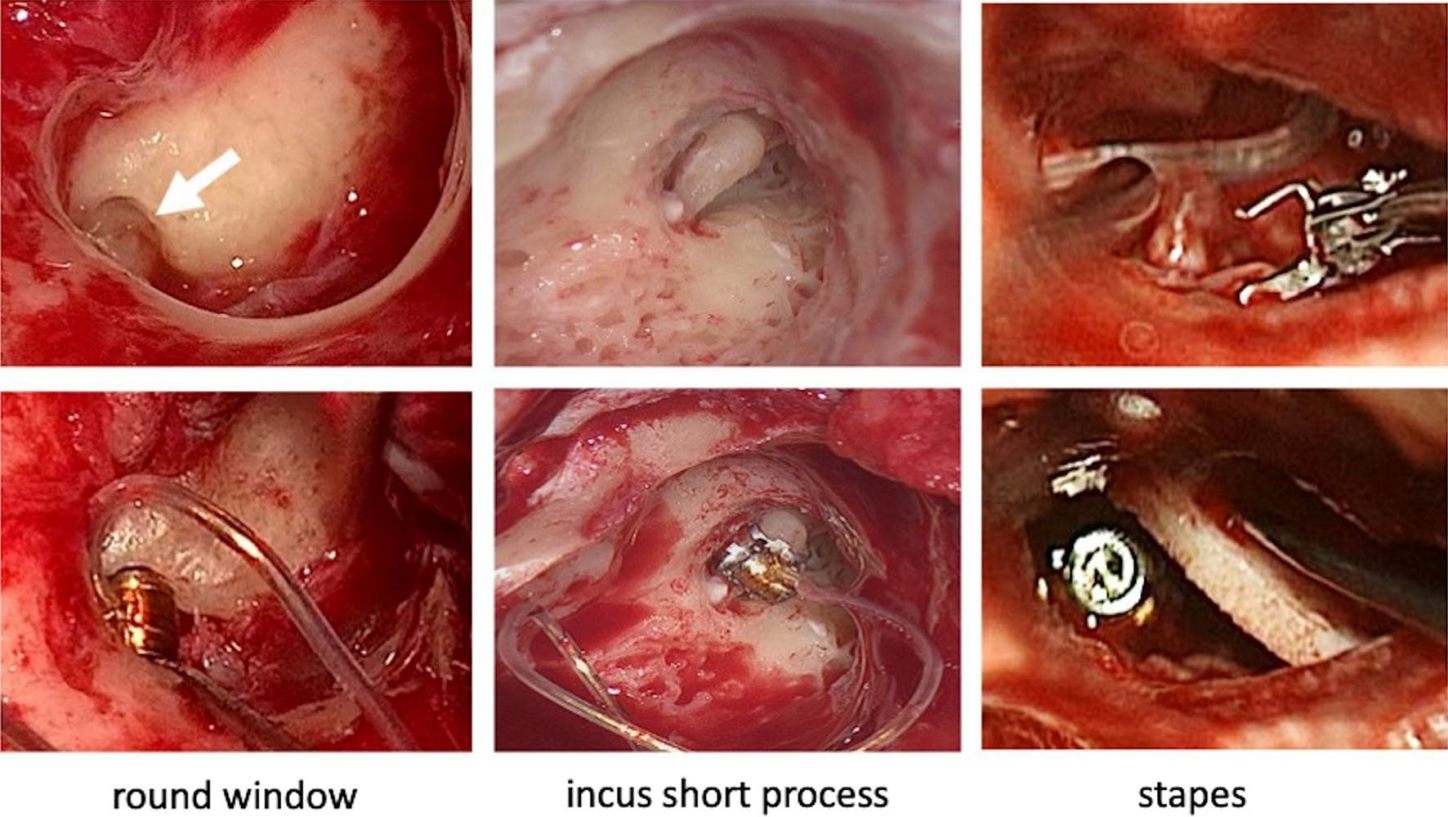
Intraoperative pictures during implantation of the Vibrant Soundbridge into the middle ear cavity. The Floating Mass Transducer (FMT) was attached (“coupling”) to the round window (RW), the short process of the incus (SP) or the stapes (St), depending on the existing middle ear structures

Each individual case was discussed with an implant board. The preoperative diagnostics included, in addition to the audiological test, a computed tomography and, if necessary, an MRI. The final decision as to which coupling system to use was based on the patient's anatomy. Depending on the extent of the previous operation, the VSB was introduced via a posterior tympanotomy, an extended antrostomy, radical cavity, or a petrosectomy [41]. The Floating Mass Transducer (FMT) in this study was attached either to the short process of the incus (SP) (*n* = 10), the stapes (St) (*n* = 18), or the round window (RW) (*n* = 14) (Table 1 and Fig. 1). Processors fitted were either the Samba (*n* = 36), or Samba 2 (*n* = 6). The reconstruction of the tympanic membrane had either already been carried out during a previous operation with tragus or conchal cartilage or was performed as part of the implantation. In cases of petrosectomy, the bony cavity was obliterated with fat, taken from the belly. The implant bed was prepared in the occipitotemporal bone and the implant was secured with an integrated screw system. Intraoperatively, the patients received a “single-shot” antibiotic prophylaxis to reduce the post-operative infection rate. The patients stayed in hospital for 2–3 nights. The bone conduction control took place on the first postoperative day, the activation of the system with good wound healing occurred after 4 weeks.

**Table 1:** Patient demographic data and medical parameters

Parameter	Summary	*n*	Age ear surgery	Age VSB implantation	Time between surgeries		Ear surgeries		
			Mean (SD)	y/m (SD)	y/m (SD)	#	Tymp	RC	Ob
n	All	42	33.1 ± 20.5	33.1 ± 20.5	4.6 ± 4.6		42	33	11
	Adults	25	46.9 ± 14.7	50.8 ± 12.9	4.4 ± 5.2		25	23	11
	Children	17	12.8 ± 3.2	16.9 ± 6.8	5 ± 4.2		17	12	0
Coupling	RW a	8	45.9 ± 10.6	50.9 ± 11.5	5 ± 4.6	1.4 ± 0.8	8	8	5
	RW c	6	14.8 ± 1.9	24.8 ± 2.8	10 ± 3	3.5 ± 0.8	6	6	0
	St a	12	43.9 ± 18	48.2 ± 15	4.5 ± 6	1.2 ± 0.2	12	10	6
	St c	6	10.3 ± 3.7	12.3 ± 3.4	2 ± 0.6	1.5 ± 0.5	6	1	0
	SP a	5	55.8 ± 9.2	56.8 ± 9.1	3.2 ± 4	1.2 ± 0.5	5	3	0
	SP c	5	13.2 ± 1.9	12.8 ± 3.8	2.6 ± 1.3	1.8 ± 1.3	5	5	0

*a* adults, *c* children, *RW* round window coupling with the round window soft coupler, *St* Stapes coupling with the vibroblasty Clip coupler, *SP* short process coupling, *#* Number of ear surgeries, *Tymp* Tymponoplasty, *RC* radical cavity, *Ob* Obliteration of the outer ear canal

### Audiological measurements

**Fig. 2:**
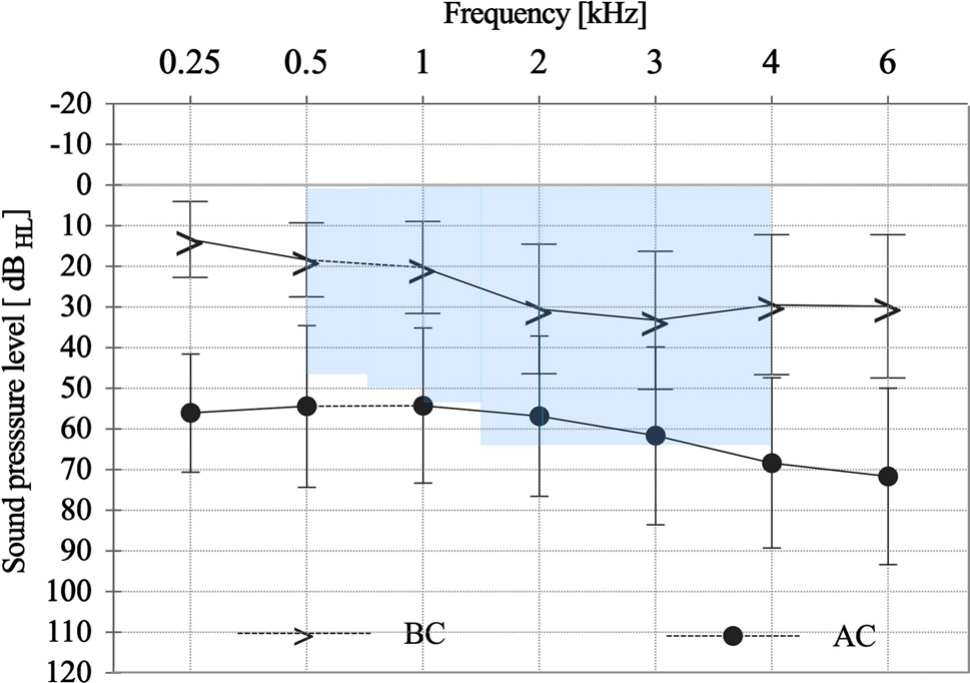
Unaided bone conduction threshold (BC) and air conduction (AC) conduction thresholds of all implanted patients. All patients are within the indication criteria (blue) for BC

Preoperative audiological measurements were performed with a clinical audiometer (Auritec AT 1000, Hamburg, Germany) in a soundproof chamber within the standardized noise level limitations, based on the International Organization for Standardization (ISO) 8253 [42]. The pure-tone audiometry and speech audiometry were performed with calibrated headphones (Beyerdynamic DT48A; Berlin, Germany) at frequencies of: 0.125, 0.25, 0.5, 1, 2, 3, 4 and 6 kHz using 5-dB increments for the VSB-implanted ear (ipsilateral) and the contralateral ear (Fig. 2), in accordance with ISO 8235–1. The averages at 0.5, 1, 2 and 4 kHz explicates the pure-tone thresholds for bone conduction (4PTA_BC_) and air conduction (4PTA_AC_). To evaluate the hearing preservation of the VSB implantation on the inner ear, the preoperative 4PTA_BC_ thresholds were compared to the last postoperative measurement. The functional gain (FG) is defined as the mean difference between unaided und aided 4PTA in the sound field (SF). To determine clinical efficiency, SF speech audiometry was performed in quiet in unaided and aided situation at 65 dB SPL (presentation from front, 0°, distance 1 m) age related to the Göttinger (children) or the Freiburger monosyllables test (adults) to receive the Word Recognition Score (WRS). The contralateral ear was plugged and covered. WRS in noise was not performed. Unaided situation was tested before implantation and in the aided situation 4 week postimplantation (i.e., activation) and 3, 6 and 12 months after activation. Values are presented as means ± standard deviations, SD.

### Aided benefit

To evaluate the benefit of the VSB intervention quantitatively, we used the subjective Glasgow Benefit Inventory (GBI) for adults, and the Glasgow Children’s Benefit Inventory (GCBI) for children. This is a patient-reported questionnaire that measures the benefit after surgery [43] and is used postoperatively in different areas of otorhinolaryngology, and was introduced by Robinson et al. in 1996 [44, 45]. The absolute total scores are finally compared and range from − 100 (maximum decline) to 0 (no benefit) to + 100 (maximum benefit). These include different subscales for adults, such as general benefit, physical benefit, and social support, and for children evaluate physical health, vitality, learning, emotion. In addition, we asked if otorrhea was present, and whether the processor is being used on a daily basis. The questionnaire was handed out once in a time-range between 6 months and 3 years after implantation.

### Sample collection and processing for microbial colonization

Patients suffering from COM were examined during their individual visits and the otological finding assessed by otoscopy pre- and postoperatively. Ear swabs were obtained preoperatively and 6 weeks postoperatively from the innermost third of the external auditory canal near the mainly perforated tympanic membrane or radical cavity from every patient, avoiding contamination from the external auditory skin. Elution-swab samples (Copan) were used for subsequent storage in Amies transport medium (Copan) and later transferred to our local microbiology laboratory, as previously described [46]. The swabs were investigated for conventional bacterial culture of commensal and pathogenic bacteria of the ear. According to the standard operating procedures, the samples were plated on blood and chocolate agar plates and incubated for up to 48 h. The numbers of swabs from the external auditory canal assessed for commensal and pathogenic bacteria, were analyzed and displayed in as percentages.

### Microbiome

Two patients were selected to obtain some insights into gut microbiome of patients with long-term suffering of severe destructive OM with multi resistant pathogens and therapy resistance over the years. The patient’s stool samples were collected in Stool Collection Tubes with DNA Stabilizer (Invitek Molecular GmbH, Berlin, Germany) and were processed using PSP^®^ Spin Stool DNA Plus Kit (Invitek Molecular GmbH, Berlin, Germany) Library preparation and Sequencing were carried out as described before [47, 48]. Bioinformatical analysis of 16S rRNA gene sequencing data was performed via mothur (version 1.44.1) [49]. Sequences were aligned against mothur’s SILVA reference database [50] and classified using Greengenes Database (49). Graphical visualization was performed using R (version 4.0.1) [51].

### Data analysis

All statistical analyses were performed using GraphPad Prism version 9.2.0. for Mac (GraphPad Software, Inc., San Diego, CA, U.S.A.). The non-parametric Wilcoxon signed-rank test was applied to evaluate significant differences between the different measurements of the WRS and the different couplings due to Shapiro–Wilk test, that showed no normal distribution. *p* values are indicated for children and adults separately, and in addition also for the total group, whereas *p* values smaller than 0.05 were considered to indicate statistical significance.

## Results

This study aimed to evaluate the audiological and microbiological outcomes of patients suffering from severe long-term OM, who received a VSB-middle ear implant surgically attached to various structures of the ME.

### Safety

**Fig. 3:**
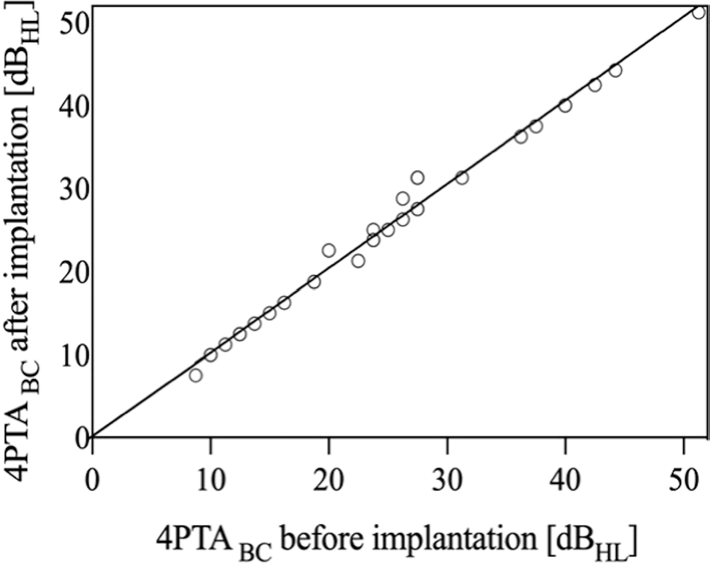
Bone conduction thresholds before (unaided) and after Vibrant Soundbridge implantation

After VSB implantation no significant decrease in hearing ability was observed, nor were there noteworthy complication rates recorded. Pre- and postoperative 4PTA_BC_ data show a net difference of 4.1 dB HL in adults (mean thresholds: preoperatively 29.1 dB HL ± 10.9; postoperatively 25 dB HL ± 10.6). In the children group, the mean VSB threshold of 2.6 dB HL (preoperative 20.6 ± 9.73; postoperative 23.2 dB HL ± 14.8) was recorded. This indicates hearing preservation after VSB surgery due to a 4PTA_BC_ change of less than 5 dB (Fig. 3) with no statistical significance (*p* = 0.163). This was independent from coupling at the short process, stapes or round window.

### Age dependent hearing benefit

To examine the possible age dependent hearing benefit association with VSB, we divided the subjects by age into adult (18 +) and children (youth under 18). The FG is 27,5 dB HL (range from 10 to 54 dB HL) with no significant (p = 0.924) difference between the adult (27,8 dB HL) and the children group (27,1 dB HL).

**Fig. 4:**
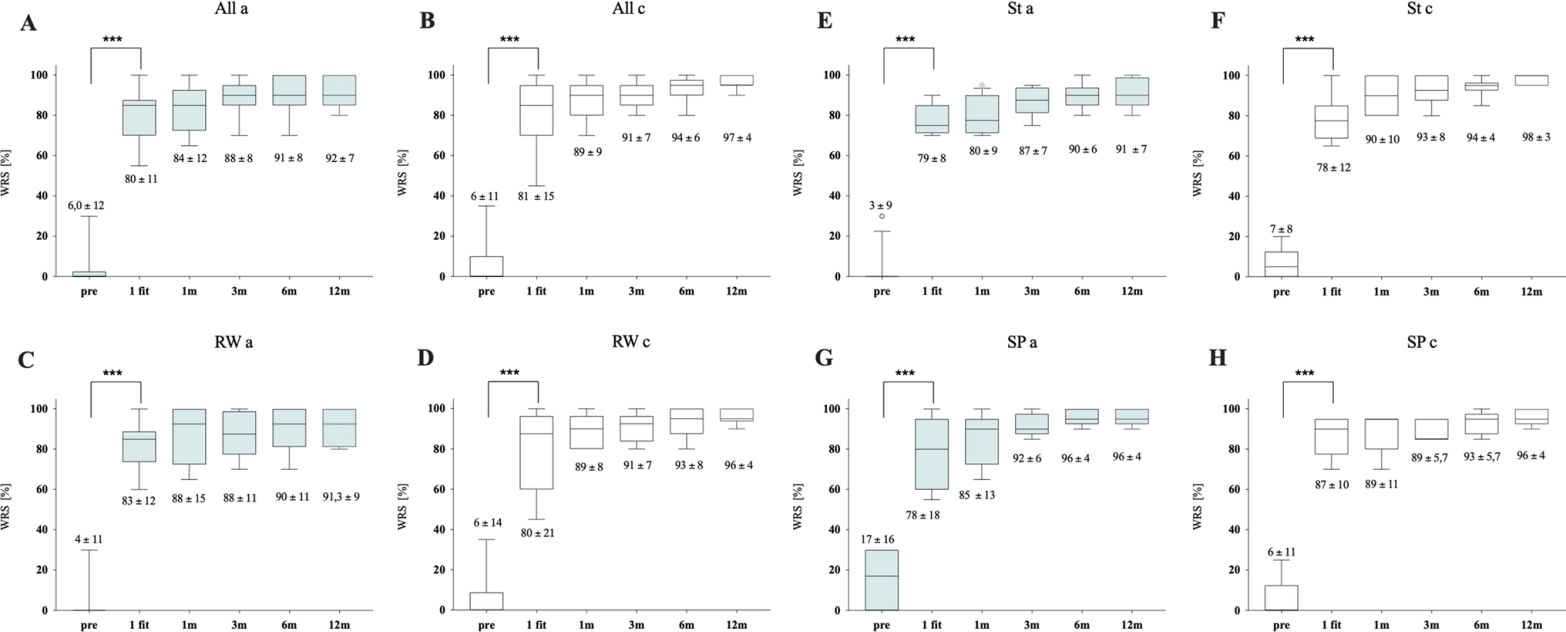
Word recognition scores (WRS) measured with the Freiburger Monosyllables represented in quiet with 65 dB SPL for **A** adults (a; *n* = 25) and **B** children (c; *n* = 17) and separated on the coupling (C–H). Preoperative (pre), activation 4 weeks after VSB implantation (1 fit), 1 month (1 m), 3 months (3 m), 6 months (6 m), 12 months (12 m) after 1 fit. RW Round window coupling (RW), St Stapes coupling (St), short process coupling (SP)

The Word recognition score (WRS) before and after implantation at 65 dB SPL in a quiet situation was used to assess the comparison between age groups. The mean unaided WRS averaged for adults was 5.95% (SD ± 12; Fig. 4A) and for children 5.88% (SD ± 11; Fig. 4B) before VSB implantation with no significant difference between the age groups (*p* = 0.8771). Pairwise comparison with the Wilcoxon signed rank test showed highly significant benefit (*p* < 0.05) in aided situation as soon as 4 weeks after surgery at the activation appointment (first fit), with WRS for adults 79.8% ± 11 (*p* < 0.0001) and for children 81.5% ± 15 (*p* < 0.0001) with no difference in between the age groups (*p* = 0.5349). The subsequent measurements (1, 3, 6 and 12 months after activation) show further gain in WRS, not statistically significant compared to the average activation data utilizing the coupling conditions: 1 month after activation (adults 83.6% ± 12 (*p* = 0.07); children 89.4% ± 9 (*p* = 0.06)), 3 months (adults 88.2% ± 8 (*p* = 0.20), children 90.9 ± 7 (*p* = 0.42)), 6 months (adults 91% ± 8 *p* = 0.07; children 93.5% ± 6 *p* = 0.21)) and 12 months [adults 92% ± 7 (*p* = 0.25), children 96.8% ± 4 (*p* = 0.07)]. There was no significant differentiation in the groups between the different coupling methods, nor within the adult and children groups compared to the WRS result at the first fit (Table 2 and Fig. 4C–H).

**Table 2:** Comparison of the Word recognition score between the different couplings within in each and to the other group

	Comparison WRS @ 65 dB SPL 1 Fit					
	Coupling in between each group			Coupling to the other group		
	RW/ST	RW/Sp	SP/St	RW	ST	SP
	*p*	*p*	*p*	*p*	*p*	*p*
Adults	0.4098	0.6425	0.933	0.8014	0.9409	0.3725
Children	0.8703	0.4939	0.2331			

*WRS* Word recognition score with Göttinger/Freiburger Monosyllables, *RW* round window, *St* stapes, *SP* Short Process of the Incus

*p* < 0.05 significant

### Benefit with VSB

**Fig. 5:**
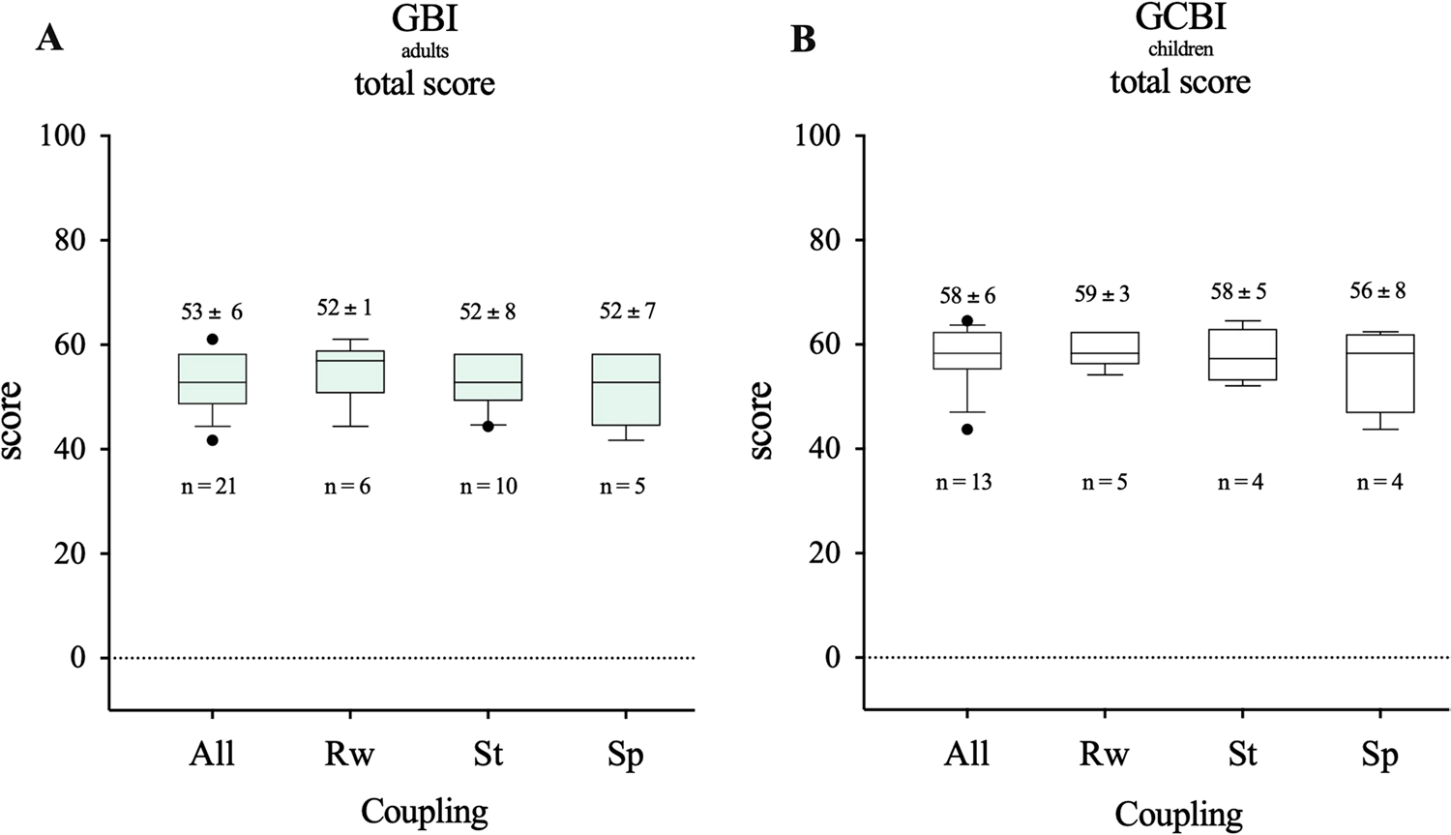
Total Score of the quality of live questionnaire for adults (**A** Glasgow Benefit Inventory; GBI) and for children (**B** Glasgow Children’s Benefit Inventory; GCBI). Scores > 0 show the benefit for all the patients in each group as well as for each coupling method. There is no significant difference in between the two groups (*p* > 0.05) and in between the coupling methods

The subjective Glasgow Benefit Inventory (GBI) for adults and Glasgow Children’s Benefit Inventory (GCBI) for children were used to assess the patient’s benefit from VSB, 84% of the adult’s group (*n* = 21) and 76% of the children’s group (*n* = 13) participated in answering the questionnaires of the GBI/GCBI. All participants showed high levels of benefit (scores > 0) after ear surgery and implantation of the VSB with a total benefit of 53 ± 6 (adults, Fig. 5) and 58 ± 6 (children, Fig. 5). There was no significant difference due the coupling method between the groups (adults: RW 52 ± 1; St 52 ± 8; Sp 52 ± 7 and children: RW 59 ± 3; St 58 ± 5; Sp 56 ± 8) or in between both groups. None of the patients suffered from significant otorrhea and every patient used the processor on a daily basis.

### Microbial colonization

**Fig. 6:**
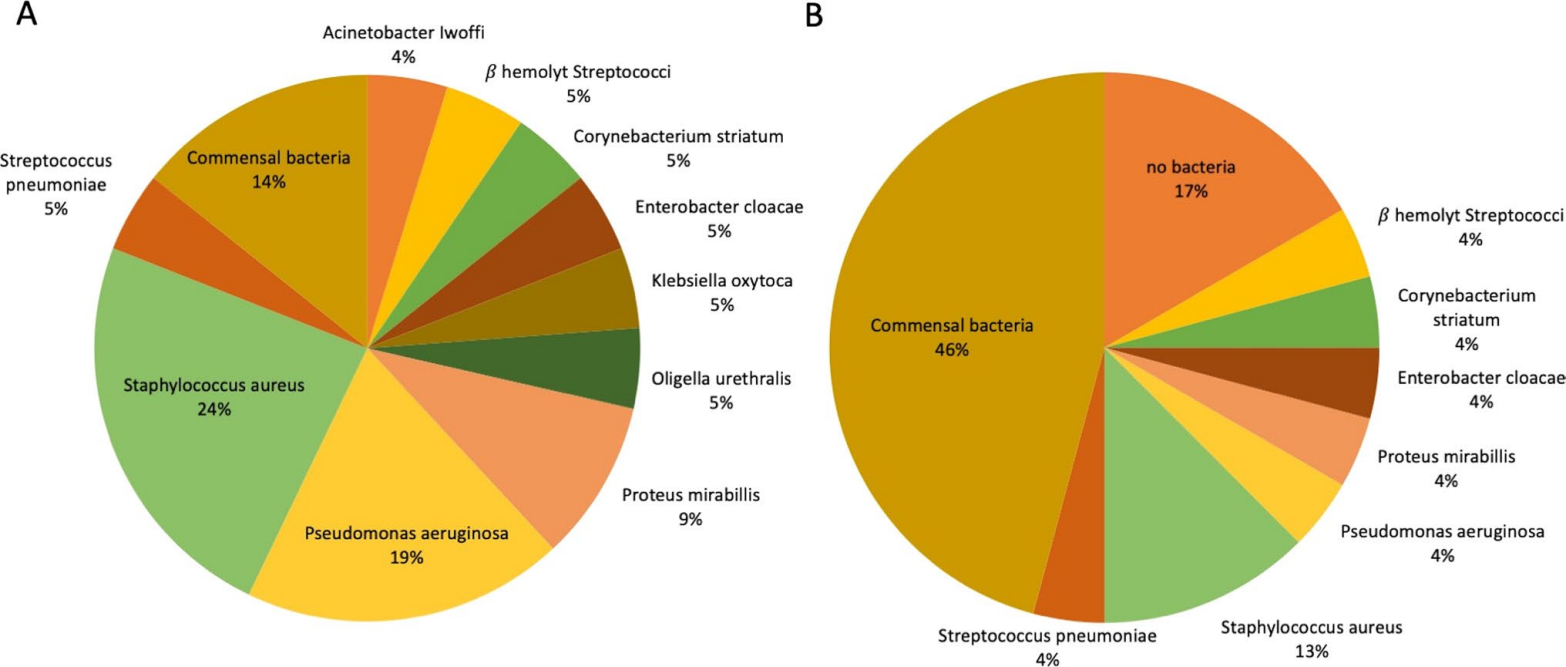
Microbial colonization in patients suffering from severe chronic Otitis media before (**A**) and after surgery und implantation of the Vibrant Soundbridge (**B**). Swabs from the external auditory canal, assessed for commensal and pathogenic bacteria, are analyzed and displayed in percentage

Presurgery, patients with severe, therapy-resistant COM display mainly two prominent pathogens: *Staphylococcus aureus* (24%) and *Pseudomonas aeruginosa* (19%), followed by *Proteus mirabilis* (9%) (Fig. 6A). Less prevalent, we found, next to *S. aureus*, more pathogens of the upper airway, such as *Streptococcus pneumonia* (5%) and *β-hemolytic Streptococci* (5%). Moreover, pathogens of the lower intestine, next to the prominent *Pseudomonas* and *Proteus* could be detected in our patients, such as *Corynebacterium striatum*, *Enterobacter cloacae* (5%) and *Klebsiella oxytoca* (5%). Moreover, ubiquitary *Acinetobacter Iwoffi* (4%) and *Origella urethralis* (5%) could be detected.

After surgery and VSB-Implantation in a one or two step procedure, perioperative antibiotic treatment and postoperative care, the microbial colonization in patients displays a distinct different pattern (Fig. 6B). After this treatment, the colonization of the ear consisted mainly of commensal bacteria (46%, compared to 14% before treatment). As particularly prevalent pathogen *Staphylococcus aureus* (13%) could be detected, whereas less prevalent, we found *Streptococcus pneumonia* (4%), β-*hemolytic Streptococci* (4%), *Pseudomonas aeruginosa* (4%), *Proteus mirabilis* (4%), *Corynebacterium striatum* (4%) and *Enterobacter cloacae* (4%), again elucidates, that many persistent inconvenient pathogens origin from the lower intestine.

### Microbiome

**Fig. 7:**
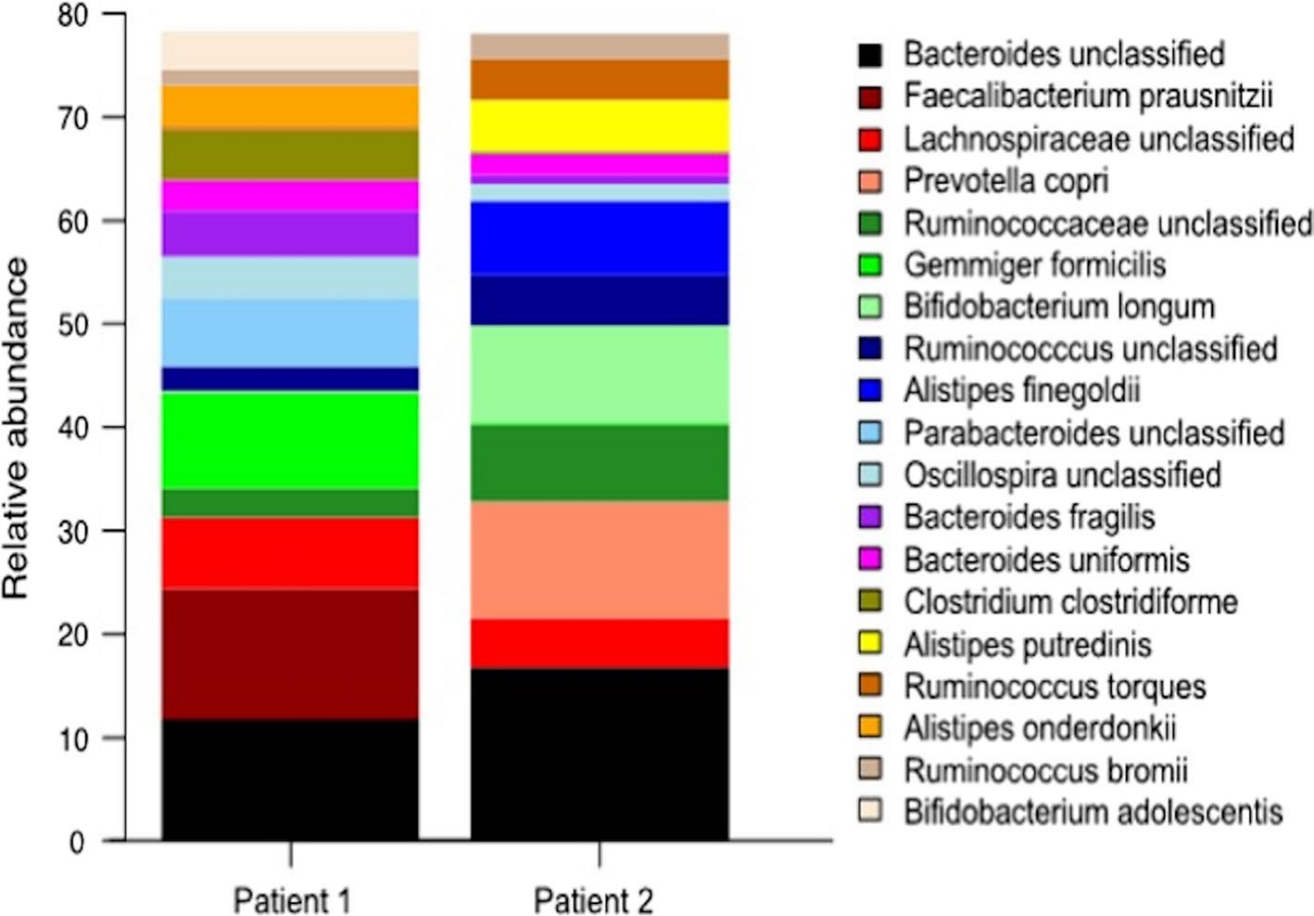
Gut microbiome composition of selected patients with known long-term multidrug resistant bacteria persistency. Relative abundance of bacterial taxa in gut microbiome prior surgery, taxa are listed ordered with more prevalent ones on top

Two of the included participants presented particular interest due to known long history of multidrug resistant *Pseudomonas aeruginosa* (patient 1) and *Proteus mirabilis* (patient 2) persistence, presenting a severe progressive destructive COM that is very difficult to deal with. Therefore, we performed gut microbiome analysis, aiming to gain a deeper understanding of the microbial composition of these selected patients. The gut consortium was dominated by phyla *Firmicutes* and *Bacteroidetes* represented by genera *Oscillospira*, *Ruminococcus*, *Gemmiger*, *Clostridium* and *Bacteroides*, *Prevotella*, *Parabacteroides*, *Alistipes* accordingly (Fig. 7). Total number of species was 79 for both samples with Shannon’s diversity index at 3.2 and Shannon’s evenness index at 0.7.

## Discussion

The causes of a chronic progressive therapy-resistant OM are multifactorial and represent a challenge in the treatment and restoration for every patient suffering from recurrent inflammation. This is often associated with chronification resulting in significant hearing impairment and otorrhea due to microbial ME infections. Moreover, wearing conventional hearing aids in the external auditory canal, often aggravate the local situation, leading to a *Circulus vicious* with recurrent, persistent otorrhea, multi-resistant pathogens, severe foetid and significant hearing impairment plus consecutive social isolation and decline. In a situation, where no conventional hearing restoration is possible due to COM, rehabilitation with an active ME (Vibrant Soundbridge) and its perioperative treatment, seems to be an elegant and effective therapy solution for both, the hearing ability and the recurrent local inflammation.

The favorable results from this study confirm the long term safety and the audiological benefit with VSB treatment [25, 39, 52, 53]. This is as well-associated with a decreased pathological bacterial colonization and a clinical anti-inflammatory effect observed in patients, who underwent multiple ear surgeries due to COM prior to VSB implantation. No major intraoperative or postoperative complications in the 42 investigated patients occurred.

The objective outcomes in terms of Word Recognition Score (WRS) showed significant improvements after surgery independent of coupling modality applied, which was also accompanied by subjective outcomes indicated by high patient satisfaction and confirm other publications for children [27, 39, 54, 55] and adults [39]. Patients with inadequate audiological rehabilitation and the resulting isolation and lack of communication appreciate the improvement und benefit with VSB.

At the same time, there was a decrease in the number of unpleasant otorrheas caused by bacterial infections. Prior to therapy, often the affected patients could not benefit from conventional hearing aids and had a tremendous lack of social interaction due to their hearing deficit. A poor fit of hearing aids resulting in recurring bacterial colonization and infections was the major reason why hearing aids were not used.

There are theories on the mismatch between normal commensal and coexisting pathogenic bacteria and, consequently, an alternate immune response that then leads to chronic infections [56]. A study by Lappan et al. compared the nasopharyngeal compositions of children, who do not suffer from OM to those with recurrent acute otitis media episodes and elaborated the hypothesis, that unaffected children might have protective commensal bacteria [57]. They found that bacteria from the genera *Corynebacterium*, *Dolosigranulum* with the species *Dolosigranulum pigrum*, and *Moraxella* with the species *Moraxella lincolnii* occurred more often in healthy children’s nasopharynx [57]. Patients with COM already underwent multiple antibiotic treatment series, that not only influence pathogens but also commensal bacteria and push mismatches forward, like those reported in acute OM [57]. A standard for a healthy ear microbiome might not be possible to determine, but knowledge about possible shielding microbes could raise the quality of COM therapy.

Pathogens in the ME are key players in the destructive process of COM and are sometimes hard to overcome. The presented patients showed a common spectrum of pathogens [58] dominated by *Staphylococcus aureus* and *Pseudomonas aeruginosa*. 10 out of the 42 patients suffered from an infection with *S. aureus*, while 8 out of the 42 had *Pseudomonas aeruginosa*, which are both well-known in COM and often develop resistance against most antibiotic remedies. Essentially, the therapy of COM and CSOM (chronic suppurative OM) face two main difficulties: first, the development of multi-resistant bacteria, e.g., 3MRGN and 4MRGN *P. aeruginosa* and, second, the formation of biofilms that allow pathogens to evade conventional antibiotic therapy [59, 60]. *S. aureus* and *P. aeruginosa* are both known to exhibit biofilms and create shielded microenvironments within the mucus layer [59]. To guarantee a satisfying outcome of COM treatment and VSB surgery, especially regarding the high benefit in the aided condition, the pathogens as well as their biofilms are needed to be eliminated before surgery. In a breakthrough, Niedzielski et al. could associate the impact of pathogens on the amount of hearing loss in a cohort of children suffering from OME [60]. The main bacteria involved were *Haemophilus influenzae*, *Streptococcus pneumoniae* and *S. aureus* and hearing loss was 10 dB worse compared to patients with negative bacterial results [60]. *S. pneumoniae* and *S. aureus* could be detected in our study population before surgery and a decrease of both species could be achieved afterward. Hence, patients have a dual benefit due to an improvement of chronic inflammation and hearing.

We were able to increase the colonization with normal commensal bacteria after surgery by more than 30% compared to pre-surgical conditions and achieved a suitable state for VSB installation. This increase of healthy commensal bacteria underlines the hypothesis that chronic inflammation is affected by a re-establishment of microbial balance. Nevertheless, 37% still presented pathogens after surgery. Noteworthy, postoperative all patients had an intact tympanic reconstruction or a fat obliterated radical cavity with a closed outer ear canal and none of the patients suffered from otorrhea. These changes might be evaluated as a possible result of the therapy as a whole including surgery and antibiotics, and further studies, investigating the microbial shift after other surgeries without VSB are necessary and ongoing.

In the end, therapeutical options are yet limited. Antibiotic treatment and aural toilet are still standard procedures to solve local infections and there is an urgent need to expand the range of possibilities and look for modulating factors beyond the aural area.

Several studies indicated a correlation between the microbial composition of upper respiratory tract and ME infection [57, 61]. A microbiome case–control study of recurrent acute otitis media identified potentially protective bacterial genera [57] as the gut–lung axis is known to play a crucial role in health and respiratory disease [62]. We selected two patients with long lasting multidrug bacteria persistence and analyzed their gut microbiome composition prior to surgery. Interestingly, several genera known to be active butyrate producers [63, 64] such as *Bacteroides*, *Bifidobacteria*, *Faecalibacterium*, *Clostridium* and *Gemminger* were highly abundant. Butyrate is a short chain fatty acid, known to play a crucial role in the modulation of anti-inflammatory responses, intestinal barrier function, [65] as well as being a key signaling compound in the gut–brain axis [66]. Still, we do not know how bacterial colonization in inflammatory conditions in the ME might be affected by intestinal microbes and their metabolic products, but the interplay of the ear–respiratory–gut axis are to be studied in more depth.

Our study is limited by its relatively small sample size. However, our observations warrant further studies with larger numbers to obtain more conclusive and deeper understanding of the microbial signaling and interactions between the respiratory tract and the intestinal microbiotic composition. It appears necessary to eliminate negative effectors of chronic inflammation, such as bacterial colonization and any probable gut–dysbiosis, to achieve a long-lasting attempt of the supplemental hearing aid systems like VSB and to avoid future re-operations. As mentioned before, non-compliance by not wearing hearing aids is higher, when there is still an unsolved inflammatory condition. Studies reported the impact of intestinal bacteria and their products on health and well-being in the context of chronic inflammation [67–69]. Particularly for patients with a complex clinical history and everyday impairment, a search for sources beyond the lines of otorhinolaryngology may be beneficial, and is subject of ongoing investigations by our group.

## Conclusion

Patients suffering from a long-term history of COM, benefit significantly from undergoing surgery and hearing rehabilitation with a Vibrant Soundbridge. This is reflected in a significant hearing improvement and benefit, including less otorrhea, pain, doctor visits and medication use, with less or no recurring OM plus a significant shift in the composition of the bacterial colonization in the ear, specifically the origin of the lower intestine. Further insights into the network of the ear–respiratory–gut axis may reveal potential probiotic candidates as well as key metabolic pathways involved in disease development.

## Data Availability

The raw sequencing data used for the microbiome analysis are publicly available at the European Nucleotide Archive (ENA) under accsession number PRJEB59100.

## Abbreviations

COM: Chronic otitis media
dB: Decibel
dB SPL: Decibel sound pressure level
dB HL: Decibel hearing level
DNA: Deoxyribonucleic acid
FG: Functional gain
FMT: Floating Mass Transducer
GBI: Glasgow Benefit Inventories
GBCI: Glasgow Benefit inventory’s for Children
HA: Hearing aid
IL: Interleukin
LPS: Lipopolysaccharides
MRI: Magnetic Resonance Imaging
MyD: Myeloid differentiation primary response
NLR: NOD-like-receptor
NOD: Nucleotide-binding oligomerization domain
OM: Otitis media
PCR: Polymerase chain reaction
PTA BC: Pure tone average bone conduction
PTA AC: Pure tone average air conduction
PORP: Partial ossicle replacement
RNA: Ribosomal ribonucleic acid
RW: Round window
SD: Standard deviation
SF: Sound field
SNHL: Sensorineural hearing loss
SP: Short process of the incus
St: Stapes
TLR: Toll-like-receptor
TNF: Tumor necrosis factor
TORP: Total ossicle replacement
VSB: Vibrant Soundbridge
WRS: Word Recognition Score
